# Development of a novel immune-related lncRNA prognostic signature for patients with hepatocellular carcinoma

**DOI:** 10.1186/s12876-022-02540-2

**Published:** 2022-11-07

**Authors:** Rui Li, Chen Jin, Weiheng Zhao, Rui Liang, Huihua Xiong

**Affiliations:** 1 Department of Oncology, Tongji Hospital, Huazhong University of Science and Technology, Wuhan, Hubei China; 2 Department of Epidemiology and Biostatistics, School of Public Health and Management, Wenzhou Medical University, Wenzhou, Zhejiang China; 3 Biological Engineering Academy, Chongqing University, Chongqing, China

**Keywords:** Hepatocellular carcinoma, Immune-related, lncRNA, Tumour microenvironment, Prognosis

## Abstract

Hepatocellular carcinoma (HCC) is the most common neoplasm and the major cause of cancer-associated death worldwide. The high mortality rate of HCC is mainly attributed to its widespread prevalence and the lack of effective treatment. Immunotherapy as a promising, innovative approach has revolutionised the treatment of solid tumours. However, owing to the heterogeneity and complex tumour microenvironment of HCC, an efficient biomarker for immunotherapy has yet to be identified. We investigated the role of immune-related long non-coding RNAs (lncRNAs) as prognostic biomarkers in patients with HCC from The Cancer Genome Atlas (TCGA) database. Spearman correlation, univariate and multivariate Cox, and lasso regression analyses were utilised to screen lncRNAs associated with prognosis. Four lncRNAs were filtered out to develop an immune-associated lncRNA prognostic signature in TCGA training as well as validation cohorts. Patients with HCC were then categorised into low- and high-risk groups according to the median value of the risk scores to evaluate the ability of the prognostic model between training and validation cohorts. A nomogram (based on risk score and stage) was constructed to appraise the general overall survival (OS) of patients with HCC. Differences in immune cell infiltration, immune checkpoint inhibitor (ICI) treatment response, gene mutation, and drug sensitivity were observed between the two groups. Thus, the lncRNA prognostic signature can serve as a sensitive prognostic biomarker with potential in individualised immunotherapy for HCC patients.

**Supplementary Information:**

The online version contains supplementary material available at 10.1186/s12876-022-02540-2.

## Introduction

Hepatocellular carcinoma (HCC) is the sixth most common malignancy worldwide and the fourth leading cause of cancer-related death [1]. Even though HCC treatment has completely improved in the past decades, approximately 30–40% of patients with HCC are generally identified at an advanced phase, with a low surgical opportunity, poor prognosis, and a five-year overall survival (OS) rate of approximately 10 to 18%. Systemic chemotherapy and targeted therapy are the main remedies for patients with advanced HCC [2]. Chemotherapy drugs for HCC include adriamycin, capecitabine, gemcitabine, oxaliplatin, and 5-fluorouracil. However, HCC has poor sensitivity to curative effects and shows side effects in response to chemotherapeutic drugs [3–5]. The multikinase inhibitor sorafenib was the first FDA-approved first-line standard effective drug that was specifically used for anti-HCC treatment in 2008 [6, 7]. It has emerged as a small-molecular inhibitor of intracellular tyrosine along with serine or threonine protein kinases (CRAF, vascular endothelial growth factor receptor [VEGFR], and BRAF), hence exhibiting the dual antitumour impact of antitumour proliferation and anti-angiogenesis. No other powerful systemic therapeutic alternative has been recognised for nearly a decade following the launch of sorafenib. However, numerous new systemic remedy alternatives have recently demonstrated efficacy in the first- and second-line settings. For example, lenvatinib is a typical first-line remedy that targets VEGFRs, fibroblast growth factor receptors, platelet-derived growth factor receptor α, RET, KIT, and stem cell factor to reduce angiogenesis and lymphoangiogenesis in HCC [8, 9]. Further second-line treatment options include regorafenib and ramucirumab besides cabozantinib as the preferred treatment regimen for HCC [10–13]. However, these remedies only elicit an increase in some months of survival, cause serious side effects, and lead to resistance within a few months. Lately, immunotherapy has achieved major revolutions in the treatment of melanoma and has paved the way for HCC [14–17]. Currently, a series of clinical trials of immunotherapy for HCC are ongoing. Immune checkpoint inhibitors (ICIs) such as nivolumab, pembrolizumab, and atezolizumab have shown promising clinical effectiveness and safety in patients with HCC. Treatment with nivolumab resulted in considerable tumour volume regression and subjective response rates of 15–20% in patients with liver cancer in the CheckMate 040 trial, regardless of lines of therapy. Furthermore, the disease control rate was 58% in the dose-escalation period and 64% in the dose-expansion period, suggesting that the OS was improved [18]. In the sub-analysis, nivolumab safety and efficacy are comparable between sorafenib-experienced intent-to-treat (ITT) and Asian patients [19]. In KEYNOTE-224, an open-label study, all patients received 200 mg pembrolizumab intravenous fluids every 3 weeks, and a 17% objective effect was observed against various risk factors linked to HCC diagnosis and treatment, including hepatitis B and C communicable disease, as well as in patients whose illnesses progressed with or who were closed-minded to sorafenib [20] [21];. Furthermore, atezolizumab combined bevacizumab exhibited a 42% lower risk of mortality and a 41% lower risk of tumour growth or death than sorafenib in the IMbrave150 study, with median progression-free survival improved by 2.5 months [22]. Immunotherapy is only 20% effective in the population owing to the complex tumour microenvironment. Extensive studies are needed to explore potentially valuable biomarkers and immune networks that are forecast of response to anti-PD-1 in addition to other remedies in terminal HCC; these studies may facilitate the identification of patients who might benefit from monotherapy and combination immunotherapy drugs [23, 24]. Long non-coding RNAs (lncRNAs) are newly discovered non-protein-coding transcripts over 200 nt in length which play a pivotal role in a broad range of biological processes and are involved in occurrence, progression, immune landscape of HCC [25–29]. So far, the diagnosis of HCC mainly relied on ultrasound imaging and alpha-fetoprotein detection, however with low sensitivity and specificity. In the research of Olga Y. Burenina et al., the expression of HELIS and LINC01093 was down-regulated and the CYTOR and HULC was up-regulated, can distinguish various hepatic malignant and benign tumors [30]. An independent study in this field found that the expression levels of lncRNA-WRAP and lncRNA-UCA1 were markedly elevated in HCC compared to those of HCV chronic infected patients or healthy individuals. When two or more lncRNA groups combined with AFP, the sensitivity and specificity of predicting the incidence of HCC were much greater than those of the simple group [31]. Further, Jinlan Huang et al. also confirmed that lncRNA panels can improve the sensitivity and specificity of HCC diagnosis. Linc00152 was observed with statistically higher levels in patients with HCC than people without malignant diseases, with an excellent performance of a single lncRNA with an AUC of 0.877. When incorporated with UCA1, AFP, this combination panel of linc00152, UCA1, and AFP had higher predictive ability and achieved an AUC value of 0.912 [32]. In addition to its diagnostic value, lncRNAs may also be potential prognostic markers in HCC. A previous study by Yufeng Wang et al. demonstrated that lncRNA MCM3AP-AS1 is a new oncogenic lncRNA that is upregulated in HCC, exerts oncogenic effects by targeting miR-194-5p, and correlates positively with tumor size, grade, stage and poor prognosis in patients with HCC [33]. Recently, a research by Gege Shu et al. showed that LINC00680 is significantly over-expressed in HCC tissues, which boosts the stemness of HCC cells and reduces the chemical sensitivity to 5-fluorouracil (5-FU) in vitro and in vivo by sponging miR-568 implying that LINC00680 may be an essential diagnostic marker and therapeutic target for HCC [34]. Thus, these findings provide a novel insights into the diagnosis and treatment of HCC and immune-related lncRNA remain to be deeply elucidated. In this study, we downloaded RNAseq data fragments per kilobase of transcript per million mapped reads (FPKM) values from The Cancer Genome Atlas (TCGA) and immune genes from the Immport database [35]. Using Spearman’s correlation valuation, 818 immunity-related lncRNAs (IRLs) were screened (*p* < 0.001, correlation coefficient ≥ 0.4). The clinical data of patients with HCC were then combined with the IRL expression matrix (excluding the patients with survival time less than or equal to 30 days, with 342 patients remaining). The remaining people were divided into a training set and a verification set. Univariate, multivariate Cox, and lasso regression analyses were employed to select IRLs linked to prognosis. Four lncRNAs were recognised to build an IRL prognostic signature. The AUC values of the training and verification sets were 0.835 and 0.822, respectively, indicating an outstanding ability of the lncRNA signature to assess prognosis. We then investigated the correlation between risk scores and clinical factors and plotted a nomogram and calibration curve. Differences in immune cell infiltration, immune checkpoint inhibitor (ICI) treatment response, gene mutation, and drug sensitivity were observed between the high- and low- risk groups. Therefore, this lncRNA prognostic signature can be used as a sensitive biomarker to predict the prognosis of patients with HCC and can positively impact personalised immunotherapy.

## Results

### HCC: detection of IRLs

**Fig. 1:**
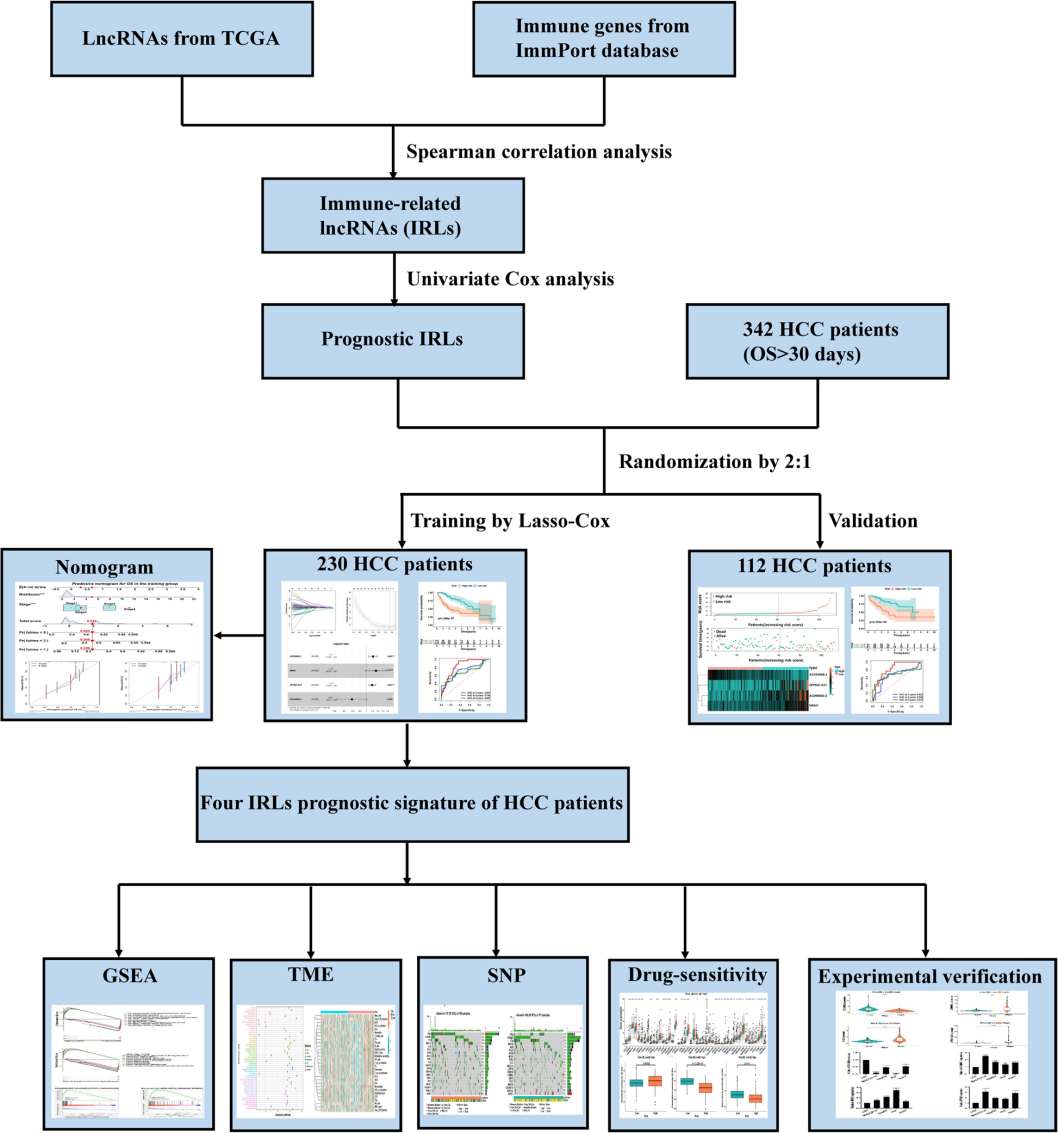
The flowchart of the present study

This study was conducted following the flowchart shown in Fig. 1. Based on the lncRNA annotation file acquired from the GENCODE, 14,143 lncRNAs from TCGA FPKM data were detected. IRLs were prescribed by an lncRNA whose expression was associated with 2046 immune genes from the Immport database, based on the criterion that the absolute value of Spearman connection coefficient was > 0.4 (*p* < 0.001). Finally, 818 IRLs were screened. The patients’ corresponding clinicopathological characteristics were then combined with the IRLs expression matrix. The 342 patients with HCC were randomly splited into training or verification sets in a 2:1 ratio for building and certifying the IRLs signature (Table 1). Notably, there was no statistically significant clinical difference between the two cohorts (Table 1, Supplementary Table 1).

**Table 1:** Clinical characteristics of hepatocellular carcinoma in train and validation cohort

Clinicopathological variables	Entire cohort	Train cohort	Validation cohort	***P***-value
	(***N*** = 342)	(***N*** = 230)	(***N*** = 112)	
**Status, N (%)**				0.504
Alive	219 (64.0)	144 (62.6)	75 (67.0)	
Dead	123 (36.0)	86 (37.4)	37 (33.0)	
**Age (years), N (%)**				0.768
≤ 65	216 (63.2)	147 (63.9)	69 (61.6)	
>65	126 (36.8)	83 (36.1)	43 (38.4)	
**Gender, N (%)**				0.961
Female	109 (31.9)	74 (32.2)	35 (31.2)	
Male	233 (68.1)	156 (67.8)	77 (68.8)	
**T-stage, N (%)**				0.412
T1	168 (49.1)	108 (47.0)	60 (53.6)	
T2	84 (24.6)	55 (23.9)	29 (25.9)	
T3	74 (21.6)	55 (23.9)	19 (17.0)	
T4	13 (3.8)	9 (3.9)	4 (3.6)	
Unknow	3 (0.9)	3 (1.3)	0 (0.0)	
**N-stage, N (%)**				0.411
N0	239 (69.9)	166 (72.2)	73 (65.2)	
N1	3 (0.9)	2 (0.9)	1 (0.9)	
Unknow	100 (29.2)	62 (27.0)	38 (33.9)	
**M-stage, N (%)**				0.453
M0	244 (71.3)	165 (71.7)	79 (70.5)	
M1	3 (0.9)	1 (0.4)	2 (1.8)	
Unknow	95 (27.8)	64 (27.8)	31 (27.7)	
**AJCC stage, N (%)**				0.213
Stage I	161 (47.1)	105 (45.7)	56 (50.0)	
Stage II	77 (22.5)	51 (22.2)	26 (23.2)	
Stage III	80 (23.4)	61 (26.5)	19 (17.0)	
Stage IV	3 (0.9)	1 (0.4)	2 (1.8)	
Unknow	21 (6.1)	12 (5.2)	9 (8.0)	
**Grade, N (%)**				0.56
G1	53 (15.5)	34 (14.8)	19 (16.9)	
G2	161 (47.1)	104 (45.2)	57 (50.9)	
G3	111 (32.5)	78 (33.9)	33 (29.5)	
G4	12 (3.5)	10 (4.3)	2 (1.8)	
Unknow	5 (1.5)	4 (1.7)	1 (0.9)	

### lncRNA prognostic signature: construction and validation

**Fig. 2:**
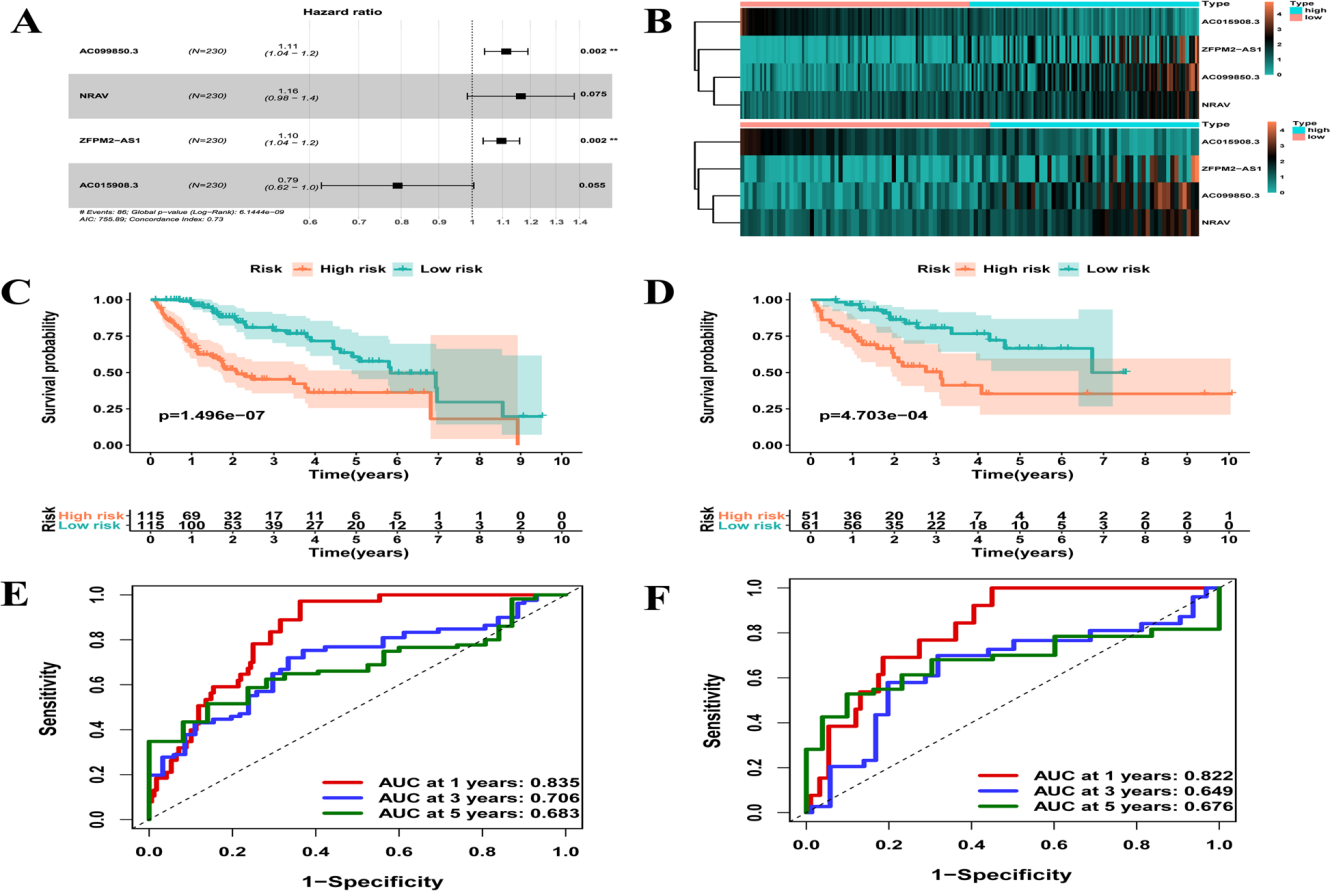
**A** Forest plots of 4 immune-related lncRNAs. **B**-**F** Distribution of heatmap, Kaplan–Meier curves and ROC curves for HCC patients in TCGA training cohort and validation cohort

A total of 76 lncRNAs were significantly related to HCC survival (*p* < 0.05) in a univariate Cox proportional exposure regression analysis (Supplementary Table 2). The best-fit OS-related lncRNAs were then identified using multivariate and LASSO regression analysis (Supplementary Fig. 1A, B). Finally, the optimum predictive hazard signature of IRLs was constructed by combining three risky lncRNAs (hazard ratio [HR] > 1) and one protective lncRNA (HR < 1). The risk score of patients with HCC was computed using the following formula: Risk score = (0.106791246 × AC099850.3) + (0.152497 × NRAV) + (0.091710183 × ZFPM2-AS1) + (0.091710183 × ZFPM2-AS1) + (0.091710183 × ZFPM2-AS1) + (0.091710183 × ZFPM2-AS1) + (0.091710183 × Z (− 0.233997454 × AC015908.3). AC099850.3, NRAV, and ZFPM2-AS1 were found as risk factors with HRs > 1, whereas AC015908.3 was identified as a protective factor with HR values < 1 (Fig. 2A). Based on the median value of the risk scores, patients were then split into low- and high-risk groups to compare the performance of the prognostic risk standard for OS estimate between the training and validation cohorts. Principal component analysis (PCA) and t-SNE analysis were first conducted to assess the clustering ability of risk signature (Supplementary Fig. 2A-D). Supplementary Fig. 1C and D describe the risk scores and survival status of patients in the two cohorts, respectively. The heatmap findings in Fig. 2B show that as hazardous lncRNAs, the expression of AC099850.3, NRAV, and ZFPM2-AS1 gains with a rising risk score, whereas AC015908.3 decreased with the increase of risk score as protective lncRNAs. The Kaplan–Meier survival analysis illustrated that patients with low risk showed a higher survival possibility than those with high risk (training cohorts: *p* < 0.001, Fig. 2C; validation cohorts: *p* < 0.001, Fig. 2D), suggesting that the risk signature of the 4 IRLs had prognostic significance. Furthermore, we assessed the risk signature’s prediction sensitivity and specificity using a time-based receiver operating characteristic (ROC) curve. For the training and validation sets, the AUC values for risk signatures at 1, 3, and 5 years were 0.835, 0.706, and 0.683 and 0.822, 0.649, and 0676, respectively (Figs. 2E, F). As a result, the potential of the four IRL signatures to forecast the prognosis of HCC was demonstrated.

### A nomogram combination risk score and Clinicopathological features predicted survival in HCC

**Fig. 3:**
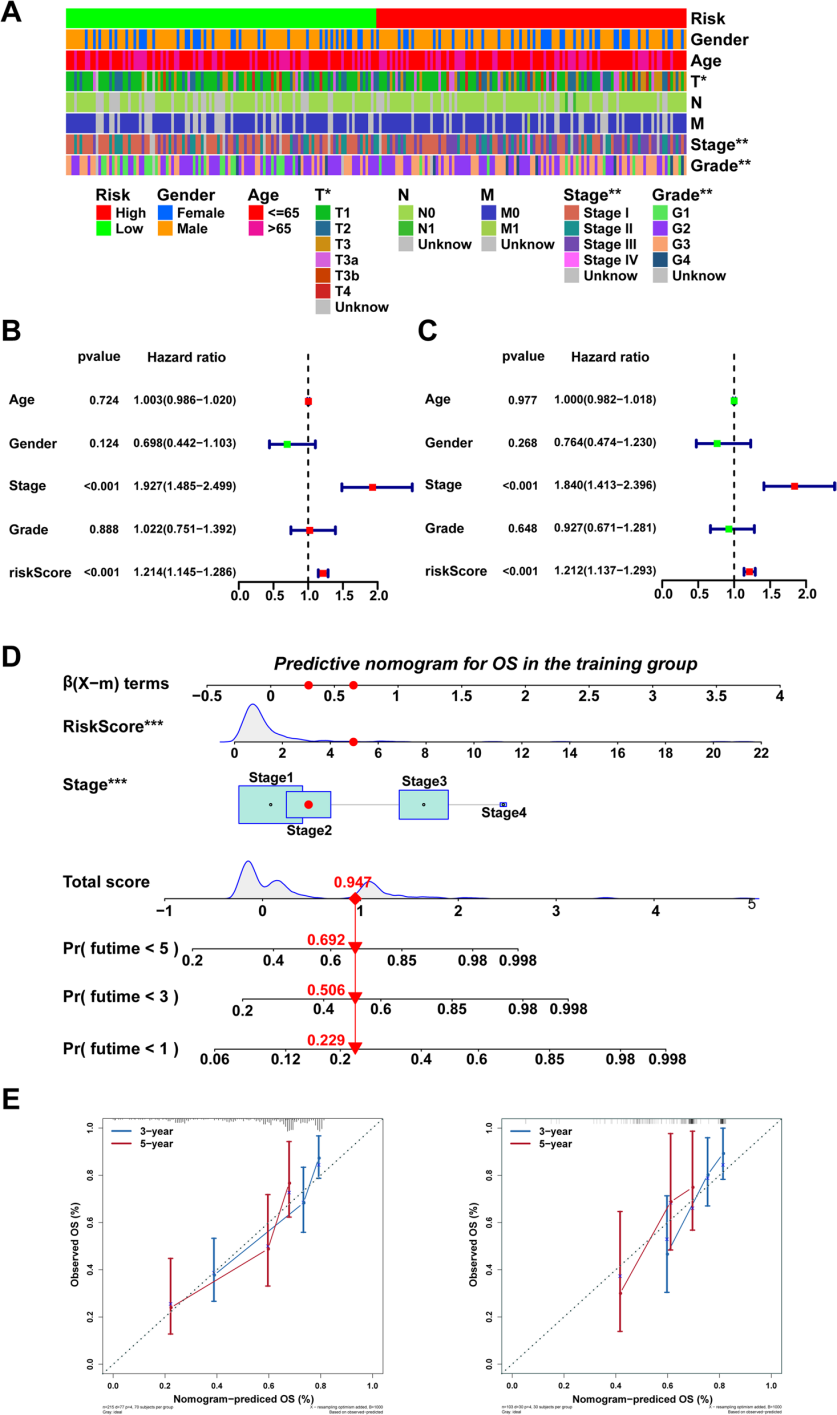
**A** Heatmap of the association between the expression levels of the 4 Immune-related lncRNAs and clinicopathological features in The Cancer Genome Atlas (TCGA) dataset. **B**, **C** Forest plots of Risk score was an independent prognostic predictor by univariate and multivariate analyses. **D** Nomogram based on risk score and clinical features. (E) Calibration plots of the nomogram for predicting the probability of OS at 3 and 5 years

The stratification assessment was performed to investigate the correlation of risk score and clinicopathological parameters in different subgroups. As shown in the heatmap (Fig. 3A), stage, T stage, and grade were significantly correlated with the risk scores (*p* < 0.05). The independence of risk signature in HCC was then assessed through univariate along with multivariate Cox regression evaluation. The risk score based on IRLs was significantly correlated with OS (HR: 1.214, 95% CI: 1.145–1.286, *p* < 0.001; Fig. 3B) in a Cox regression univariate assessment. Furthermore, multivariate Cox regression assessment revealed that the lncRNA risk score may predict HCC prognosis individually (HR: 1.212, 95% CI: 1.137–1.293, *p* < 0.001; Fig. 3C). By utilising all independent prognostic variables discovered through multivariate Cox regression analysis, a nomogram based on the IRLs was developed to investigate the survival probability of 1-, 3-, and 5-year survivors (Fig. 3D). When the overall score was 0.947, the corresponding 1-year, 3-year, and 5-year survival probabilities were 0.771, 0.494, and 0.308, respectively, as shown in Fig. 3D. The nomogram calibration curves were drawn to determine the predicted survival rates and observed survival probabilities (Fig. 3E).

### Gene set enrichment analyses (GSEA)

**Fig. 4:**
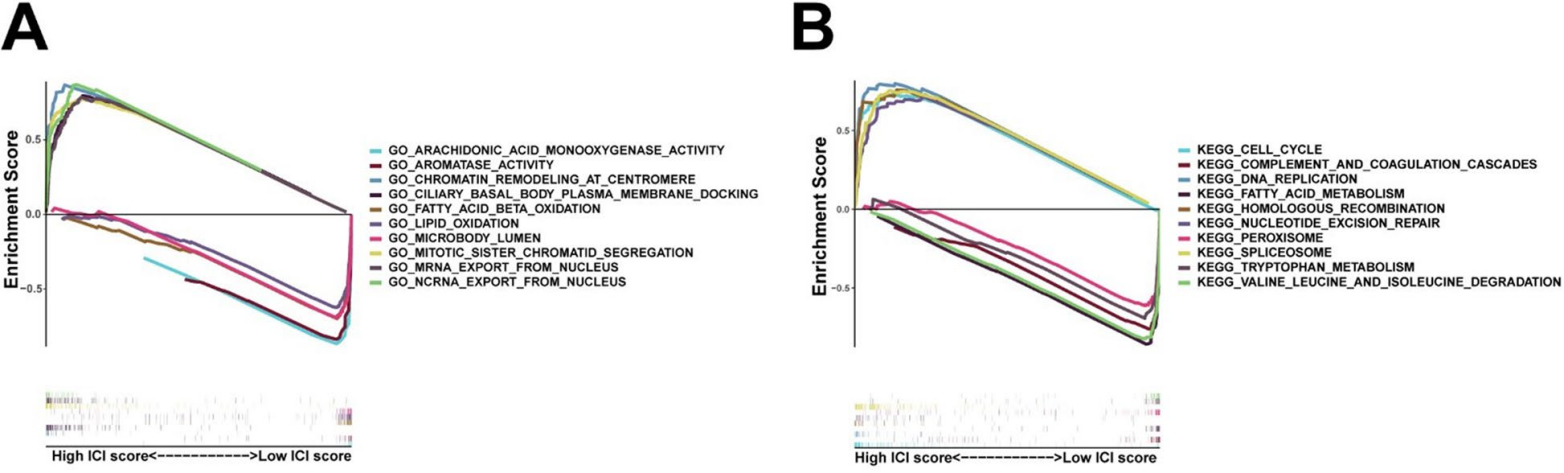
**A**-**D** Gene set enrichment analysis (GSEA) based on risk score showed the relevance between biological process, molecular function, cellular component and KEGG

We performed GSEA based on the risk scores to better understand the underlying mechanisms related to the lncRNA prognostic signature in patients with HCC. The GO terms were enriched mainly in arachidonic acid monooxygenase activity, aromatase activity, chromatin remodelling at centromere, ciliary basal body plasma membrane docking, fatty acid beta oxidation, lipid oxidation, microbody lumen, mitotic sister chromatid segregation, mRNA export from the nucleus, and ncRNA export from the nucleus (NES) (Fig. 4A). The KEGG pathway enrichment analysis (Fig. 4B) revealed that the prognostic signature was significantly related to cell cycle, complement and coagulation cascades, DNA replication, fatty acid metabolism, homologous recombination, nucleotide excision repair, peroxisome, spliceosome, tryptophan metabolism, and valine, leucine, and isoleucine degradation.

### Risk scores correlated with tumour microenvironment, single-nucleotide polymorphisms (SNPs), and drug sensitivity

**Fig. 5:**
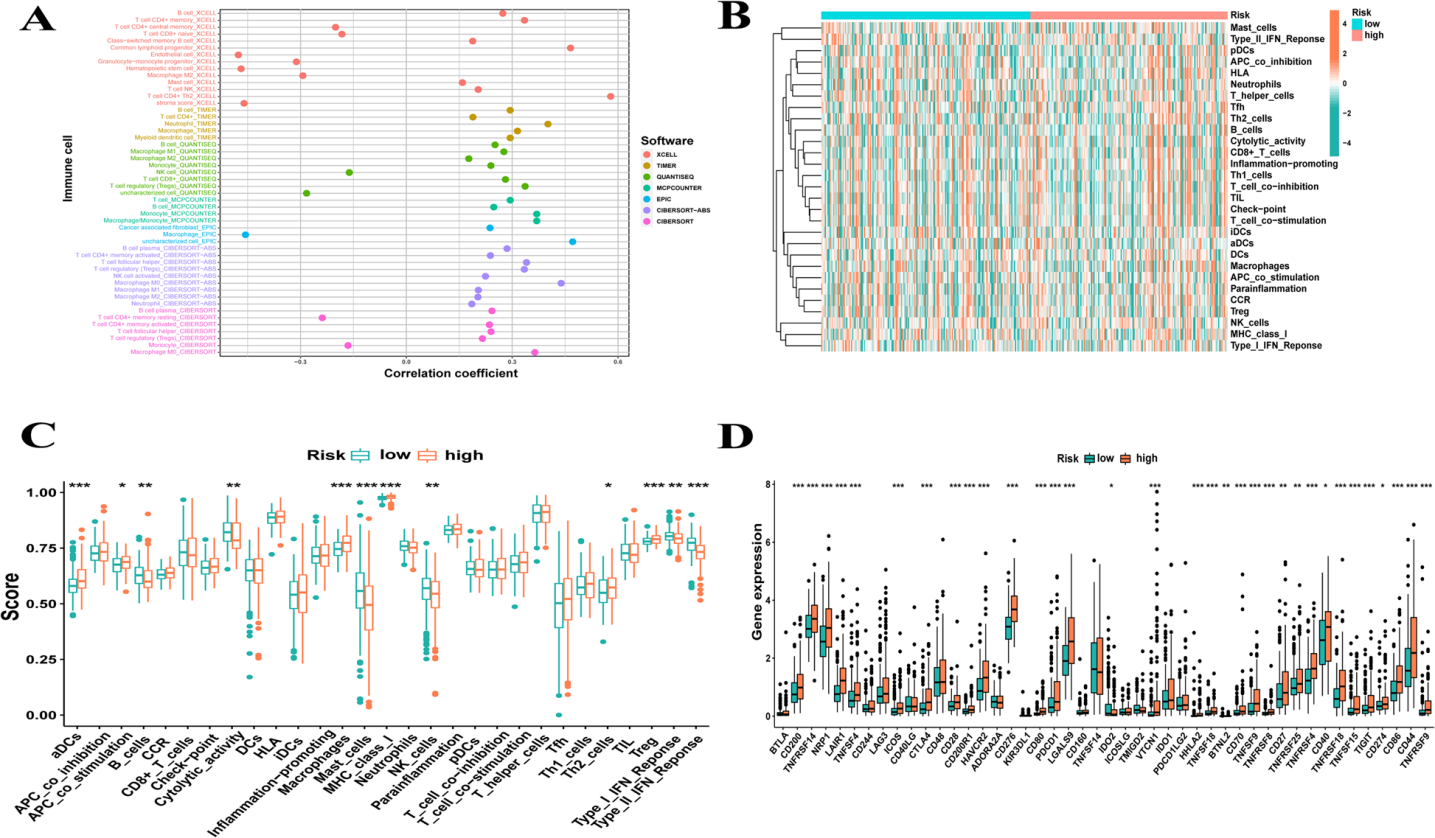
**A** Seven methods assess the immune cell infiltration including XCELL, TIMER, QUANTISEQ, MCPCOUNTER, EPIC, CIBERSORT-ABS and CIBERSORT. **B**, **C** The immune immune cells and pathways quantify by ssGSEA, shown by heat map and boxplot in high−/low-risk group. **D** The expression level of possible immune checkpoints in high−/low-risk group in TCGA cohort

**Fig. 6:**
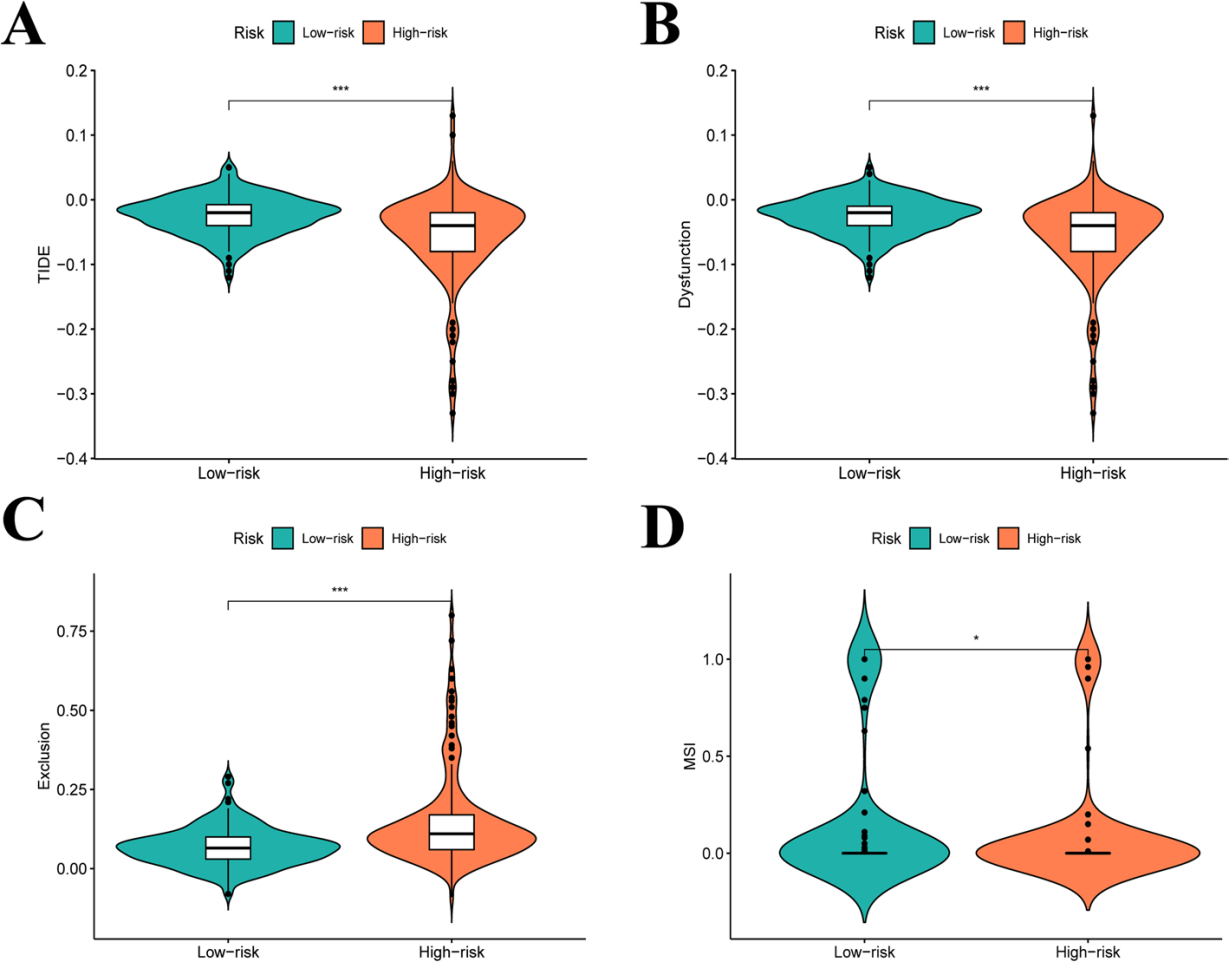
**A**-**D** TIDE score distribution in the high- and low-risk groups

**Fig. 7:**
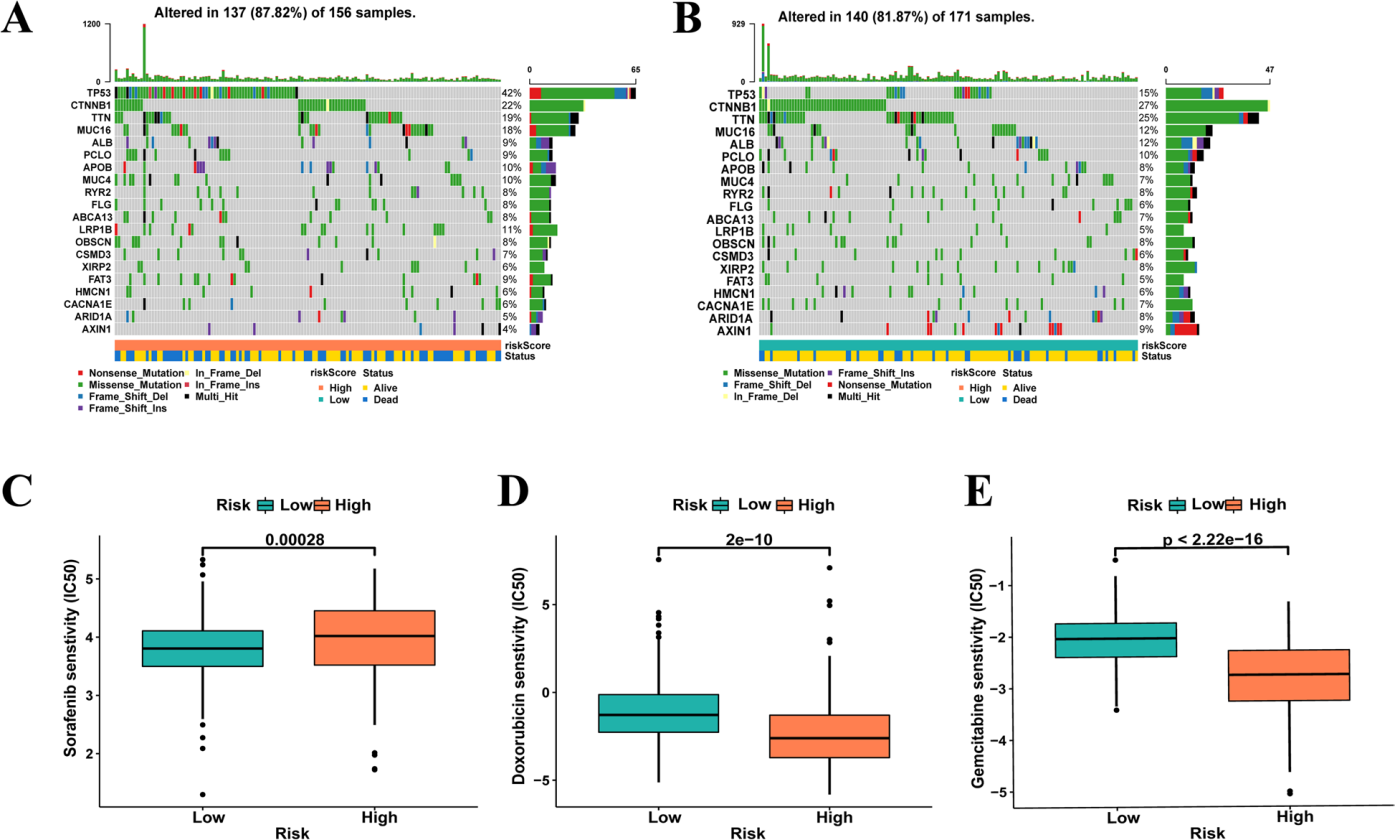
**A**, **B** Waterfall maps of twenty mutated genes in high−/low-risk group. **C**-**E** The relationship between targeted therapy, chemotherapy and IC50 in high−/low-risk group

We applied seven common suitable methods to appraise the immune cell infiltration, including MCPCOUNTER, XCELL, TIMER, QUANTISEQ, CIBERSORT-ABS, EPIC, and CIBERSORT (Fig. 5A). Positive correlation coefficients were broadly observed, indicating that patients with higher risk scores were experiencing immunological activation. Most immune cells identified using XCELL, such as CD4+ Th2 cells, were positively associated with the risk score. Meanwhile, neutrophils in TIMER, regulatory T cells (Tregs) in QUANTISEQ, monocytes in MCPCOUNTER, and M0 macrophages in CIBERSORT-ABS and CIBERSORT also showed positive correlations. Besides, endothelial cells in XCELL, uncharacterised cells in QUANTISEQ, macrophage in EPIC, CD4+ resting memory T cells in CIBERSORT were negatively correlated with the risk score; infiltration of these cells indicated an immunosuppression condition in the HCC high-risk group. In addition, single sample GSEA (ssGSEA) was used to assess the immune cells and pathways involved in HCC. The heatmap in Fig. 5B reveals the relationship between HCC risk, immune cells, and pathways. The boxplot in Fig. 5C demonstrated that aDCs, APC_co_stimulation, macrophages, MHC_class_I, Th2_cells, and Tregs were exceedingly expressed in the group with high risk, whereas B_cells, cytolytic_activity, mast_cells, NK_cells, type_I_IFN_response, and type_II_IFN_response were highly expressed in the group with low risk (*p* < 0.05). Tumor immune dysfunction and exclusion (TIDE) was developed based on the two main mechanisms of tumor immune escape by Jiang et al. [36] which can predict the ICI treatment response. Therefore, we measured the scores of TIDE, Dysfunction, Exclusion and MSI in each HCC patient to predict clinical response to immunotherapy based on the IRLs signature. As shown in Fig. 6A-D**,** TIDE, Dysfunction and MSI were highly expressed in the low risk group, while Exclusion was the opposite (*p* < 0.05). Furthermore, we analysed the relationship between possible the immune checkpoints and risk signature. Figure 5D indicates that our risk model is associated with the expression of most immune checkpoints, with PDCD1 (PD-1), CD274 (PD-L1), and CTLA4 being highly expressed in the high-risk group. In the risk model, the condition of SNPs was also investigated. Among the 156 patients with high risk, 137 (87.82%) had mutated genes. In the high-risk group, TP53 accounted for 42% of all mutations, a remarkably higher value than that in the group with low risk (Fig. 7A). In the group with low risk, genes were altered in 140 (81.87%) of 171 samples (Fig. 7B). *CTNNB1, TTN*, and *AXIN1* accounted for 27, 25, and 9% of all mutations, respectively, which are slightly higher values than those observed in the high-risk group. Finally, the relationship between risk score and clinical drug sensitivity was analysed. As shown in Fig. 7C, sorafenib sensitivity was positively associated with the high-risk group, whereas doxorubicin (Fig. 7D) and gemcitabine (Fig. 7E) were highly sensitive in the low-risk cluster.

### LncRNA expression in risk signature and experimental verification

**Fig. 8:**
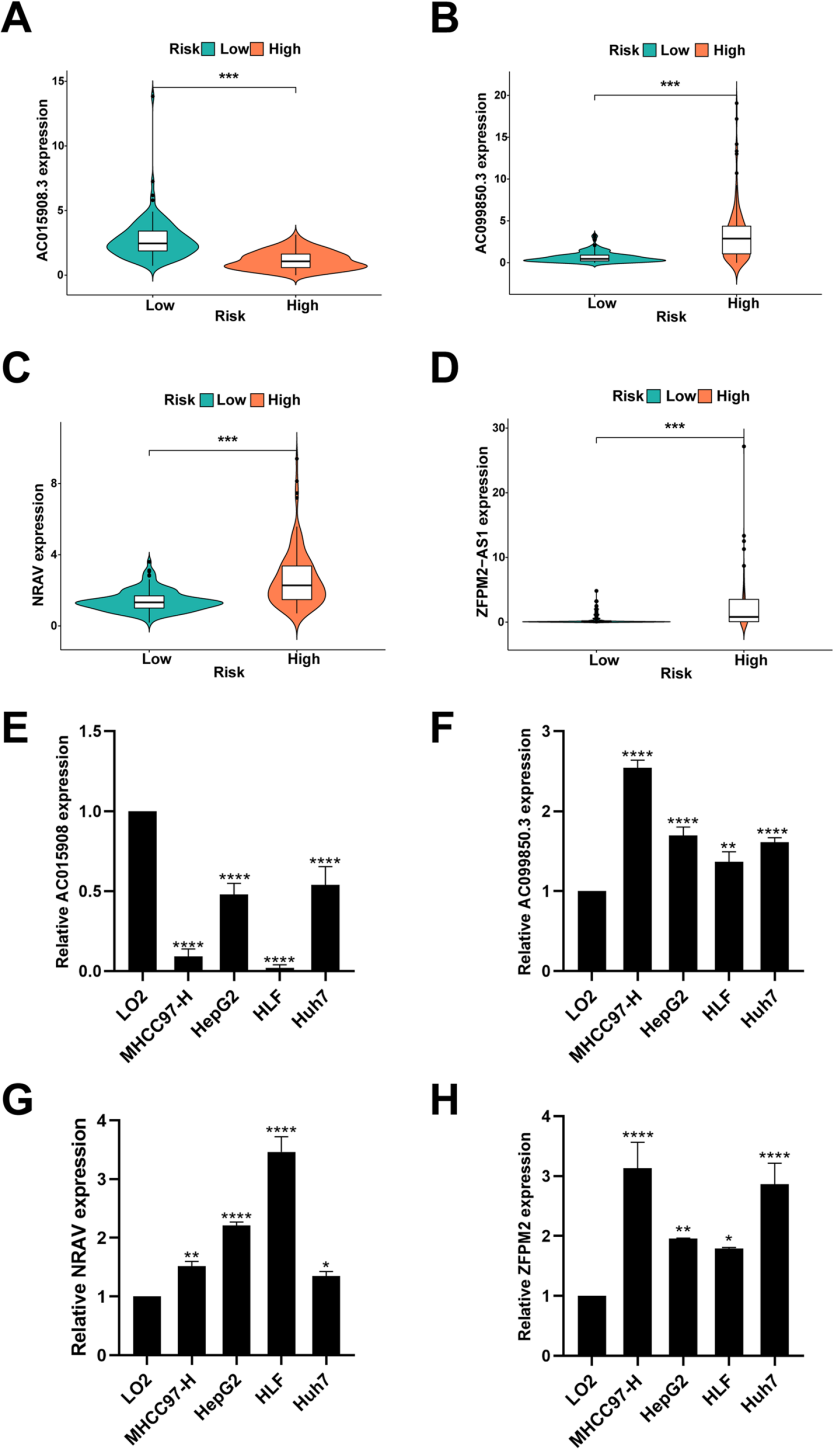
**A**-**D** The expression of 4 immnue-related lncRNA in high−/low-risk group. **E**-**H** Experimental verification in normal liver cells and liver cancer cells

We explored the lncRNA expression in the high- and low-risk groups. As shown in Figs. 8A–D, AC015908.3 expression was upregulated in the low-risk group, whereas AC099850.3, NRAV, and ZFPM2-AS1 were highly expressed in the high-risk group (*P* < 0.05). We then verified the expression level of lncRNA in normal liver cell (LO2) and liver cancer cells (MHCC-97 h, HepG2, HLF, and Huh7). As shown in Figs. 8E–H, AC015908.3 expression was downregulated in HCC cells, while AC099850.3, NRAV, and ZFPM2-AS1 was upregulated in HCC cells compared to normal liver cell (*P* < 0.05).

## Discussion

HCC is a highly common malevolent tumour with a poor prognosis. There have been tremendous advances in the treatment of HCC over several decades [5, 37]. Nevertheless, these provides only minor prolongation of general survival and a marginal boost in quality of life. Immunotherapy is considered the revolutionary breakthrough of cancer treatment, shifting the focus from the tumour to the tumour microenvironment and has been notably successful for the cure of melanoma coupled with non-small cell lung cancer; this achievement laid the foundation for immunotherapy for HCC [38]. In 2018, nivolumab and pembrolizumab received accelerated FDA approval for second-line medication in patients with HCC. Based on the results of the IMBrave 150 trial, the FDA approved the combination strategy of atezolizumab (anti-PDL1 antibody) and bevacizumab (anti-VEDF antibody) as the first-line treatment for unresectable HCC in 2020 [22, 39, 40]. However, the response rates of patients with HCC to immune checkpoint inhibition remain low (~ 15–20%) and strongly dependent on the tumour microenvironment. Thus, more efforts are needed to explore biomarkers that predict patient survival or the efficacy of immunotherapy [39, 40]. Recent studies have demonstrated that lncRNAs play crucial roles in cancer immunity [41]. For example, lnc-CENDE can promote the M2 polarisation of macrophages and regulate tumour angiogenesis [42] and Lnc-Tim3 promotes CD8+ T-cell exhaustion [43]. In our study, to recognise potential prognostic biomarkers and explore the role of the tumour microenvironment in HCC, we evaluated the data from TCGA dataset through bioinformatics analyses. First, 818 IRLs were filtered from TCGA RNAseq data through Spearman correlation analysis. Through univariate, multivariate cox, and LASSO regression analyses, four lncRNAs constituted the optimal prognostic risk signature of IRLs, namely AC099850.3, NRAV, AC015908.3, and ZFPM2-AS1. Patients were divided into low- and high-risk groups between the training and validation cohorts based on median risk scores to assess the performance of this prognosis risk model. The group with low risk had a better OS than the group with high risk. In addition, the AUC values further confirm the predictive sensitivity and specificity of the risk signature. Among these features, NRAV is expressed in various human cells and plays an important role as a regulatory molecule by negatively regulating the expression of some crucial antiviral proteins [44]. Zhou’s study indicated that NARV was overexpressed in human HCC cell lines [45], consistent with our RT-qPCR results. AC099850.3 were overexpressed, promoting HCC cells to migrate and proliferate in vitro, and might have upregulated the expression of cell cycle-related markers such as CDK1, PLK1, BUB1, and TTK. Furthermore, AC099850.3 was associated with the T-cell receptor signalling cascade, which affects the expression of CD155 along with PD-L1 in HCC cell lines [46]. The lncRNA ZFPM2-AS1, an upregulated lncRNA in HCC, acts as a miRNA sponge in HCC and promotes cell invasion by regulating miR-139/GDF10. In addition, ZFPM2-AS1 indicates a poor prognosis and leads to HCC progression via the miR-653/GOLM1 axis. Reportedly, ZFPM2-AS1 may act as a prospective therapeutic target and prognostic biomarker for HCC [47]. Furthermore, AC015908.3 is closely related to cancer cell stemness and prognosis [48, 49]. Conversely, in our study, AC015908.3 was found to be overexpressed in tumour cell lines and associated with immunity. A nomogram combining risk scores and clinicopathological parameters predicting prognosis in HCC was constructed, and the calibration curves were plotted to estimate the predicted survival probability. We also performed GSEA based on risk scores to better understand the mechanisms of the IRL prognostic signature in patients with HCC. As ICIs have been used for treating terminal HCC, we explored the relationship between TIDE, ICI-related biomarkers and the risk signature to predict clinical response to immunotherapy. These results indicate that high-risk patients may have a better response to immunotherapy. Besides, immune cells and associated inflammatory responses in the tumour microenvironment can influence the response to anti-checkpoint blockades. For example, myeloid-derived suppressor cells contribute to a tumour immunosuppressive microenvironment and immune-checkpoint blockade resistance. Tumour-infiltrating lymphocytes, CD8+ T cells, and NK cells are associated with anti-PD1 immunotherapy. We used seven widely accepted techniques to quantify immune cell infiltration to examine the association between risk signature and tumour-infiltrating immune cells. The results showed that CD4+ Th2 cells, neutrophils, Tregs, monocytes, and M0 macrophages were positively correlated with the risk score, whereas endothelial cells, uncharacterised cells, macrophages, and CD4+ resting memory T cells were inversely correlated with the risk score. Furthermore, we utilised ssGSEA to assess the immune cells and pathways. Patients with low risk scores had high infiltration levels of B_cells, mast_cells, NK_cells, while aDCs, macrophages, Th2_cells, and Tregs were highly expressed in the high-risk group. As for immune-related pathways, checkpoint type_I_IFN_response, and type_II_IFN_response were negatively correlated with risk score, while APC_co_stimulation and MHC_class_I were positively correlated with risk score. In view of the large number of tumour mutation-derived neoantigens that can activate the immune system and affect the efficacy of anti-checkpoint blockade. We then estimated the condition of SNPs in the risk model. In the high-risk group, *TP53* accounted for 42%, a remarkably higher value than that in the group with low risk, consistent with Calderaro’s research, wherein *TP53*-mutated HCC cells were poorly differentiated, densely packed, highly proliferative, multinucleated, and pleomorphic and exhibited frequent vascular infiltration [50]. In the low-risk group, *CTNNB1* was the most mutated gene, with slightly higher expression than that in the high-risk group. *CTNNB1* mutations characterise a particular cholestatic well-differentiated subtype of HCC [50, 51]. Finally, we analysed the correlation between risk and the effectiveness of common therapies such as chemotherapy and targeted treatment in HCC. We discovered that high risk was correlated with a significantly high half-maximal inhibitory concentration (IC50) of sorafenib (*p* < 0.001) but a lower IC50 for chemotherapy drugs such as doxorubicin (*p* < 0.001) and gemcitabine (*p* < 0.001), signifying that our model could be used to predict chemotherapy and targeted therapy sensitivity. However, it is undeniable that there are some limitations to this study. The model was established and validated only using TCGA data, with no external validation from the Gene Expression Omnibus (GEO) or other databases; thus, the final model may be inaccurate. However, various methods were used to verify this novel prognostic risk signature, revealing its superior potential. As a result, we believe that our model is admissible. In the future, we will collect tissue samples, increase the sample size, and perform multi-centre validation to enhance the predictability of the model; nevertheless, these further studies will be expensive and time-consuming. In conclusion, our research developed a new predictive signature derived from IRLs and assessed the role of tumor microenvironment in patients with HCC. The signature may help identify individuals with HCC who could benefit from anticancer immunotherapy and offer possible targets for accurate prediction.

## Materials and methods

### Datasets

RNAseq information of carcinoma and adjacent tissues in patients with HCC and relevant clinical data were achieved from TCGA website (https://portal.gdc.cancer.gov/). Data of the immune genes were downloaded from the ImmPort database (https://www.immport.org/home). The KEGG pathway enrichment analysis was accomplished from the KEGG pathway database [52–54] (https://www.kegg.jp/kegg/kegg1.html). In addition, the somatic mutation records of patients with HCC were acquired with a mutation annotation system (MAF) file from TCGA. The data of immune cell content in TCGA HCC patients were acquired in TIMER2.0 (http://timer.cistrome.org).

### Bioinformatics analysis

First, ‘TCGAbiolinks’ R package was used to download RNASeq and corresponding clinical data. The ensemble human genome browser GRCh38.p13 was employed to distinguish lncRNAs from protein-coding genes (Cunningham et al., 2019). The somatic mutation database of patients with HCC was obtained from TCGA using an MAF file, and the mutation data were visualised using the ‘maftools’ platform in R software. Using the Caret R package, 342 patients were randomised to either the training or verification cohorts in a 2:1 ratio. The IRLs were then extracted using Spearman’s correlation analysis, and 818 IRLs were filtered out. We used univariate and multivariate Cox regression assessment and LASSO regression analysis to identify the greatest-fit OS-related lncRNAs. Finally, four lncRNAs were found to be the optimised predictive risk signatures, and the scoring system for patients with HCC was derived using the following formula: Risk score = (1.1 × AC099850.3) + (1.16 × NRAV) + (1.10 × ZFPM2-AS1) + (− 0.79 × AC015908.3). The OS predictive performance of the prognostic risk model was evaluated between the training and validation cohorts by dividing patients into low- and high-risk groups based on median risk scores. We used GSEA to better understand the mechanism underlying the novel lncRNA prognostic signature in patients with HCC through R packages “clusterProfiler”, “enrichplot” and “ggplot2”. The gene sets “c5.go.v7.4.symbols.gmt” and “c2.cp.kegg.v7.4.symbols.gmt” were chosen as the reference gene set. Simultaneously, we also utilised the ssGSEA technique to quantify immune cells and processes involved in the mechanism. IC50 was calculated using the R package pRRophetic, and the IC50 in high- and low-risk groups was evaluated employing the Wilcoxon signed-rank test. Spearman correlation analysis was also used to analyse the relationship between the risk score, immune checkpoint, and drug sensitivity. The ggplot2 R platform (Wickham, 2016) was utilised for visualisation.

### Cell culture

The normal hepatic cell (LO2) and liver tumour cells (HepG2, MHCC-97 h, HLF, and Huh7) were kindly provided by liver surgery laboratory of Tongji. LO2 cells were cultured on 1640 medium (containing 10% foetal bovine serum), and the other cell lines were cultured on DMEM. Cell culture was performed under standard cell culture conditions in humidified 5% CO_2_.

### RT-qPCR

RT-qPCR was performed on an ABI 7900 qPCR system using ChamQ Universal SYBR qPCR Master Mix reagent (Vazyme), following the manufacturer’s instructions. The primers used for RT-qPCR were synthesised by Tsingke Biological (Supplementary Table 3). Each reaction was carried out three times, and data were analysed using the 2-ΔΔCT method, using *GAPDH* as an internal reference.

### Statistical analyses

For clinical data analysis, the chi-squared test or Fisher’s precise test was employed. The IRLs were discovered using Spearman correlation analysis. We used Cox regression univariate, multivariate, and lasso regression evaluation to find the best-fit OS-related lncRNAs. The Kaplan–Meier method and log-rank test were used for survival analysis between the high- and low-risk groups. The sensitivity and specificity of risk profile prediction were assessed using time-dependent ROC curves. Stratification analysis was conducted to explore the correlation between the risk characteristics and clinicopathological characteristics in different subgroups. The Wilcox test was used to compare the proportion of tumour-infiltrating immune cells, immune checkpoint molecule expression level, and drug sensitivity between the high and low-risk groups. R (version 4.0.3) and the associated packages were used for all computational and statistical studies. Two-tailed *p* values < 0.05 were considered statistically significant.

## Supplementary Information

**Additional file 1: Table 1.** Clinical characteristics of hepatocellular carcinoma in train and validation cohort.

**Additional file 2: Supplementary Table 2.** 76 prognostic associated lncRNAs.

**Additional file 3: Supplementary Figure 1.** (A) The best-fit OS-related lncRNAs were chosen by Lasso regression analysis. (B) The Lasso regression was performed with the optimal value of λ. (C-D) Distribution of risk scores, survival status. **Supplementary Figure 2.** (A-D) PCA among all genes, immune genes, immune LncRNA, and risk immune LncRNA.

**Additional file 4: Supplementary Table 3.** All the primer sequences.

## Data Availability

The information from TCGA is widely available (https://www.cancer.gov/tcga), and the current research followed TCGA data access policies and publication guidelines.
