# Supplementary material for: Development of a novel immune-related lncRNA prognostic signature for patients with hepatocellular carcinoma
Source: BMC Gastroenterol. 2022 Nov 7;22:450. doi: 10.1186/s12876-022-02540-2 (PMC9639314; doi:10.1186/s12876-022-02540-2)
Supplement: Supplementary file 1 — Additional file 1: Table 1. Clinical characteristics of hepatocellular carcinoma in train and validation cohort. [file 12876_2022_2540_MOESM1_ESM.docx]

**Table 1 |** Clinical characteristics of hepatocellular carcinoma in train and validation cohort

| **Clinicopathological variables** | **Entire cohort** | **Train cohort** | **Validation cohort** | **P-value** |
| --- | --- | --- | --- | --- |
|  | **(N=342)** | **(N=230)** | **(N=112)** |  |
| **Status, N (%)** |  |  |  | 0.504 |
| Alive | 219 (64.0) | 144 (62.6) | 75 (67.0) |  |
| Dead | 123 (36.0) | 86 (37.4) | 37 (33.0) |  |
| **Age(years), N (%)** |  |  |  | 0.768 |
| ≤65 | 216 (63.2) | 147 (63.9) | 69 (61.6) |  |
| ＞65 | 126 (36.8) | 83 (36.1) | 43 (38.4) |  |
| **Gender, N (%)** |  |  |  | 0.961 |
| Female | 109 (31.9) | 74 (32.2) | 35 (31.2) |  |
| Male | 233 (68.1) | 156 (67.8) | 77 (68.8) |  |
| **T-stage, N (%)** |  |  |  | 0.412 |
| T1 | 168 (49.1) | 108 (47.0) | 60 (53.6) |  |
| T2 | 84 (24.6) | 55 (23.9) | 29 (25.9) |  |
| T3 | 74 (21.6) | 55 (23.9) | 19 (17.0) |  |
| T4 | 13 (3.8) | 9 (3.9) | 4 (3.6) |  |
| Unknow | 3 (0.9) | 3 (1.3) | 0 (0.0) |  |
| **N-stage, N (%)** |  |  |  | 0.411 |
| N0 | 239 (69.9) | 166 (72.2) | 73 (65.2) |  |
| N1 | 3 (0.9) | 2 (0.9) | 1 (0.9) |  |
| Unknow | 100 (29.2) | 62 (27.0) | 38 (33.9) |  |
| **M-stage, N (%)** |  |  |  | 0.453 |
| M0 | 244 (71.3) | 165 (71.7) | 79 (70.5) |  |
| M1 | 3 (0.9) | 1 (0.4) | 2 (1.8) |  |
| Unknow | 95 (27.8) | 64 (27.8) | 31 (27.7) |  |
| **AJCC stage, N (%)** |  |  |  | 0.213 |
| Stage I | 161 (47.1) | 105 (45.7) | 56 (50.0) |  |
| Stage II | 77 (22.5) | 51 (22.2) | 26 (23.2) |  |
| Stage III | 80 (23.4) | 61 (26.5) | 19 (17.0) |  |
| Stage IV | 3 (0.9) | 1 (0.4) | 2 (1.8) |  |
| Unknow | 21 (6.1) | 12 (5.2) | 9 (8.0) |  |
| **Grade, N (%)** |  |  |  | 0.56 |
| G1 | 53 (15.5) | 34 (14.8) | 19 (16.9) |  |
| G2 | 161 (47.1) | 104 (45.2) | 57 (50.9) |  |
| G3 | 111 (32.5) | 78 (33.9) | 33 (29.5) |  |
| G4 | 12 (3.5) | 10 (4.3) | 2 (1.8) |  |
| Unknow  **Fustat N (%)**  Alive  Dead  **Adjacent.hepatic.tissue.**  **inflammation.extent.type N (%)**  Mild  None  Severe  Unknown  **Child.pugh.classification.**  **grade N (%)**  A  B  C  Unknown  **Fibrosis.ishak.score N (%)**  0 - No Fibrosis  1,2 - Portal Fibrosis  3,4 - Fibrous Speta  5 - Nodular Formation and Incomplete Cirrhosis  6 - Established Cirrhosis  **Residual.tumor N (%)**  R0  R1  R2  Unknown  **Race N (%)**  AMERICAN INDIAN OR ALASKA NATIVE  ASIAN  BLACK OR AFRICAN AMERICAN  Unknown  WHITE  **Height (mean (SD))**  **Weiht (mean (SD))**  **BMI (mean (SD))**  **Albumin.result.specified.value (mean (SD))**  **Prothrombin.time (mean (SD))** | 5 (1.5)  219 (64.0)  123 (36.0)  93 (27.2)  112 (32.7)  17 (5.0)  120 (35.1)    204 (59.6)  20 (5.8)  1 (0.3)  117 (34.2)  72 (21.1)  30 (8.8)  24 (7.0)  8 (2.3)  65 (19.0)  143 (41.8)  302 (88.3)  15 (4.4)  1 (0.3)  24 (7.0)  1 (0.3)  148 (43.3)  15 (4.4)  10 (2.9)  168 (49.1)  167.66 (9.11)  73.06 (19.10)  25.89 (6.08)  22.94 (310.54)    4.11 (4.97) | 4 (1.7)  114 (62.6)  86 (37.4)  57 (24.8)  73 (31.7)  14 (6.1)  86 (37.4)  113 (57.8)  13 (5.7)  1 (0.4)  83 (36.1)  46 (20.0)  21 (9.1)  15 (6.5)  4 (1.7)  42 (18.3)  102 (44.3)  201 (87.4)  12 (5.2)  0 (0.0)  17 (7.4)  1 (0.4)  102 (44.3)  6 (2.6)  8 (3.5)  113 (49.1)  167.96 (9.51)  72.38 (17.82)  25.55 (5.48)  32.65 (384.11)  4.16 (5.18) | 1 (0.9)  37 (33.0)  39 (34.8)  3 (2.7)  34 (30.4)  7 (6.2)  0 (0.0)  34 (30.4)  9 (8.0)  9 (8.0)  4 (3.6)  23 (20.5)  41 (36.6)  3 (2.7)  1 (0.9)  7 (6.2)  46 (41.1)  9 (8.0)  2 (1.8)  55 (49.1) | 0.504  0.209  0.649  0.687  0.338  0.167  0.406  0.355  0.164  0.473  0.819 |
