# Supplementary material for: Development of a novel immune-related lncRNA prognostic signature for patients with hepatocellular carcinoma
Source: BMC Gastroenterol. 2022 Nov 7;22:450. doi: 10.1186/s12876-022-02540-2 (PMC9639314; doi:10.1186/s12876-022-02540-2)
Supplement: Supplementary file 2 — Additional file 2: Supplementary Table 2. 76 prognostic associated lncRNAs. [file 12876_2022_2540_MOESM2_ESM.docx]

**Supplementary table 2 | 76 prognostic associated lncRNAs.**

| **Gene Symbol** | **HR (95%CI)** | **P-Value** |
| --- | --- | --- |
| AC099850.3 | 1.174 (1.112,1.241) | 1.01209E-08 |
| AL031985.3 | 2.329 (1.694,3.202) | 1.93076E-07 |
| ZFPM2-AS1 | 1.134 (1.079,1.192) | 7.31709E-07 |
| NRAV | 1.386 (1.218,1.578) | 7.74251E-07 |
| AC145207.5 | 1.938 (1.475,2.545) | 2.01269E-06 |
| SNHG3 | 1.08 (1.043,1.118) | 1.25362E-05 |
| LINC01138 | 1.427 (1.216,1.673) | 1.2879E-05 |
| AL117336.3 | 1.598 (1.293,1.976) | 1.45267E-05 |
| AC015908.3 | 0.608 (0.482,0.765) | 2.35944E-05 |
| NCK1-DT | 1.71 (1.326,2.204) | 3.47511E-05 |
| SNHG10 | 1.39 (1.188,1.626) | 4.05085E-05 |
| AC026401.3 | 1.104 (1.053,1.158) | 4.46272E-05 |
| LINC00623 | 1.443 (1.203,1.729) | 7.42856E-05 |
| AC006504.8 | 1.513 (1.232,1.859) | 7.85185E-05 |
| LINC00942 | 1.036 (1.018,1.055) | 9.19433E-05 |
| AC110285.6 | 1.48 (1.214,1.806) | 0.000109328 |
| PIK3CD-AS2 | 1.337 (1.154,1.549) | 0.00011247 |
| AC006026.3 | 1.608 (1.263,2.049) | 0.000118597 |
| KCNMB2-AS1 | 1.288 (1.132,1.464) | 0.000118824 |
| AC010761.1 | 1.561 (1.228,1.984) | 0.000270455 |
| AC068987.4 | 1.154 (1.067,1.247) | 0.000306684 |
| THUMPD3-AS1 | 1.383 (1.158,1.653) | 0.000351191 |
| ZNF674-AS1 | 1.739 (1.282,2.359) | 0.000372004 |
| DANCR | 1.031 (1.014,1.049) | 0.000421822 |
| AL365203.2 | 1.125 (1.054,1.201) | 0.000425517 |
| AC083799.1 | 1.13 (1.055,1.209) | 0.000446252 |
| AC016735.1 | 1.045 (1.02,1.071) | 0.000462628 |
| FOXD2-AS1 | 1.235 (1.097,1.389) | 0.000467154 |
| AC124798.1 | 1.236 (1.097,1.392) | 0.00050279 |
| AL603839.3 | 1.404 (1.157,1.705) | 0.000605233 |
| LINC00221 | 1.159 (1.065,1.262) | 0.000629323 |
| TMEM220-AS1 | 0.728 (0.606,0.875) | 0.000696941 |
| RBPMS-AS1 | 1.623 (1.226,2.148) | 0.000705097 |
| AC016710.1 | 1.182 (1.073,1.303) | 0.000754102 |
| WAC-AS1 | 1.104 (1.042,1.169) | 0.000764071 |
| LINC01273 | 1.357 (1.135,1.623) | 0.000796695 |
| AC068473.5 | 1.475 (1.172,1.856) | 0.000914206 |
| SNHG14 | 1.369 (1.134,1.651) | 0.001059425 |
| AC004816.1 | 1.222 (1.082,1.38) | 0.001199423 |
| CASC19 | 1.145 (1.055,1.242) | 0.001201653 |
| AC016394.1 | 1.355 (1.126,1.632) | 0.00133349 |
| AC023157.3 | 1.236 (1.085,1.408) | 0.001402567 |
| AL109613.1 | 1.233 (1.084,1.402) | 0.001432871 |
| C8orf49 | 1.486 (1.161,1.901) | 0.001656438 |
| MIR210HG | 1.133 (1.047,1.225) | 0.001988101 |
| AC009779.2 | 1.174 (1.06,1.3) | 0.002085594 |
| CTD-2297D10.2 | 1.106 (1.037,1.179) | 0.002096781 |
| SNHG12 | 1.156 (1.052,1.272) | 0.002676777 |
| PWAR6 | 1.17 (1.054,1.299) | 0.003258526 |
| EGFR-AS1 | 1.174 (1.054,1.306) | 0.003370605 |
| SNHG21 | 1.693 (1.19,2.407) | 0.003387462 |
| ZEB1-AS1 | 1.445 (1.129,1.848) | 0.003424565 |
| AC010969.2 | 1.389 (1.113,1.732) | 0.003606102 |
| SBF2-AS1 | 1.545 (1.151,2.073) | 0.003797433 |
| AC040970.1 | 1.259 (1.077,1.472) | 0.003878916 |
| AC074117.1 | 1.46 (1.129,1.888) | 0.003955191 |
| AC012615.1 | 1.294 (1.085,1.544) | 0.004071939 |
| AC138207.5 | 1.128 (1.039,1.225) | 0.004236708 |
| BACE1-AS | 1.185 (1.055,1.331) | 0.004242978 |
| AC005586.2 | 1.234 (1.068,1.426) | 0.00438973 |
| LUCAT1 | 1.261 (1.075,1.479) | 0.00444722 |
| AC079305.1 | 1.03 (1.009,1.051) | 0.004677522 |
| AC145343.1 | 1.328 (1.09,1.618) | 0.004949694 |
| LINC02001 | 1.087 (1.025,1.152) | 0.005188535 |
| AL049840.4 | 1.086 (1.025,1.15) | 0.005215815 |
| LINC01436 | 1.082 (1.023,1.144) | 0.005599864 |
| SNHG7 | 1.037 (1.01,1.064) | 0.006889966 |
| AC016747.1 | 1.199 (1.051,1.368) | 0.006930473 |
| MSC-AS1 | 1.194 (1.049,1.358) | 0.007096553 |
| ITGB1-DT | 1.095 (1.025,1.17) | 0.007099269 |
| SNHG1 | 1.04 (1.011,1.071) | 0.007365229 |
| AC092687.3 | 1.234 (1.055,1.444) | 0.008398974 |
| NIFK-AS1 | 1.322 (1.074,1.628) | 0.00850699 |
| AC012467.2 | 1.495 (1.104,2.024) | 0.009347411 |
| GAS5 | 1.007 (1.002,1.012) | 0.009868816 |
| AC004656.1 | 1.185 (1.042,1.349) | 0.009898702 |
