# Supplementary material for: Development of a novel immune-related lncRNA prognostic signature for patients with hepatocellular carcinoma
Source: BMC Gastroenterol. 2022 Nov 7;22:450. doi: 10.1186/s12876-022-02540-2 (PMC9639314; doi:10.1186/s12876-022-02540-2)
Supplement: Supplementary file 3 — Additional file 3: Supplementary Figure 1. (A) The best-fit OS-related lncRNAs were chosen by Lasso regression analysis. (B) The Lasso regression was performed with the optimal value of λ. (C-D) Distribution of risk scores, survival status. Supplementary Figure 2. (A-D) PCA among all genes, immune genes, immune LncRNA, and risk immune LncRNA. [file 12876_2022_2540_MOESM3_ESM.docx]

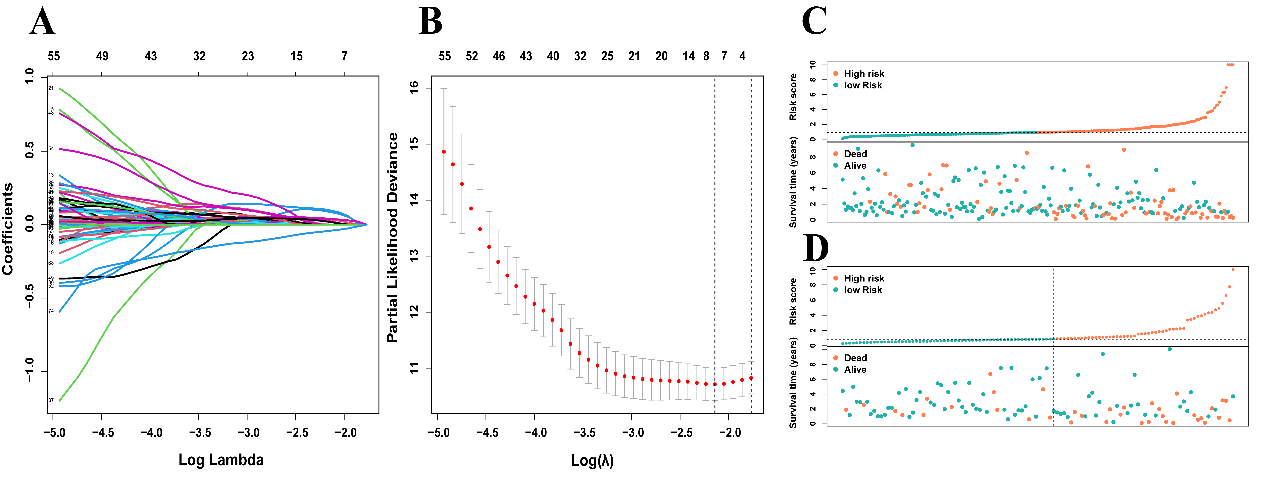


**Supplementary Figure 1 |** **(A) The best-fit OS-related lncRNAs were chosen by Lasso regression analysis. (B) The Lasso regression was performed with the optimal value of λ. (C-D) Distribution of risk scores, survival status.**


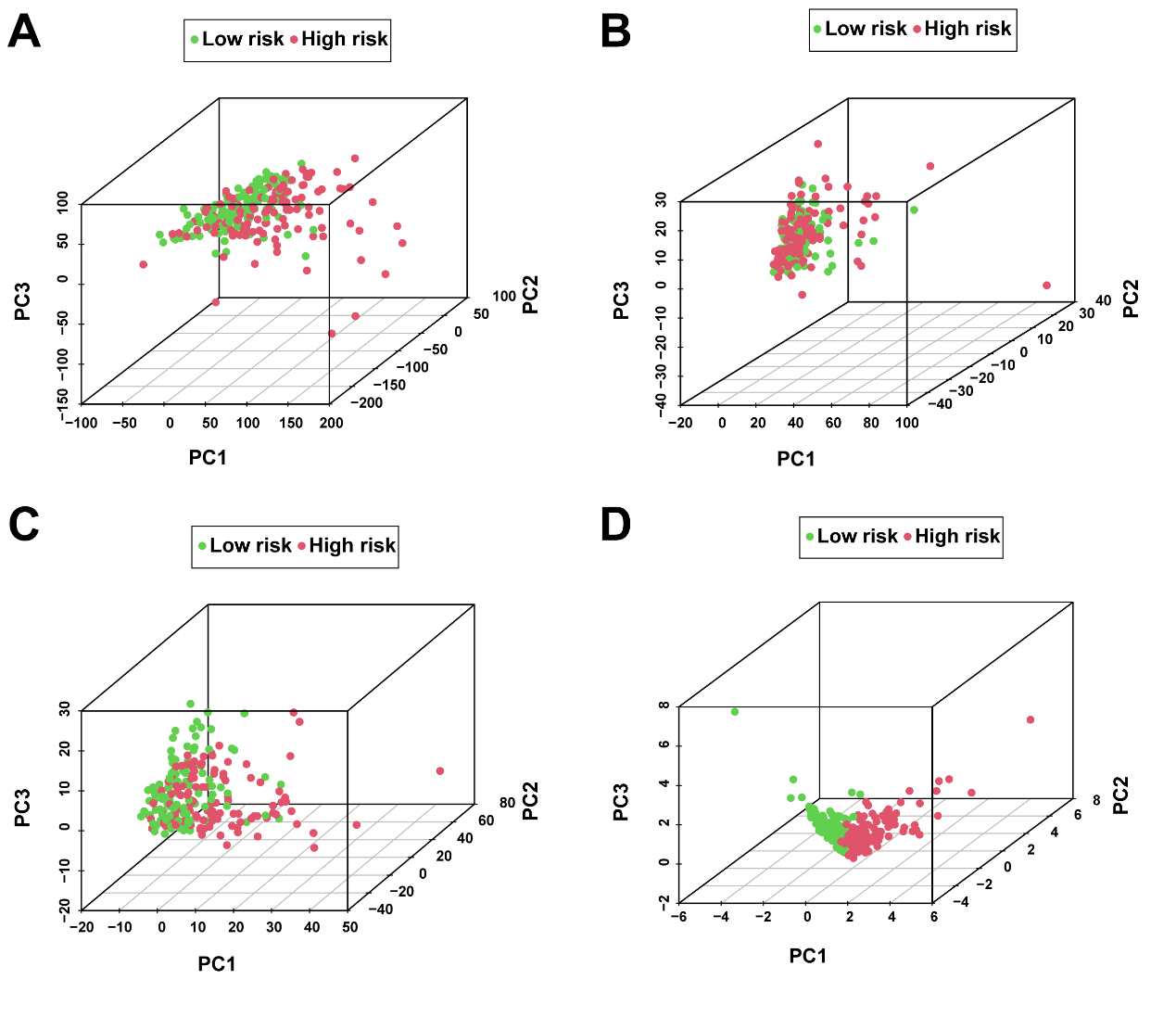


**Supplementary Figure 2 |** **(A-D)** PCA among all genes, immune genes, immune LncRNA, and risk immune LncRNA.
