# Supplementary material for: Development of a novel immune-related lncRNA prognostic signature for patients with hepatocellular carcinoma
Source: BMC Gastroenterol. 2022 Nov 7;22:450. doi: 10.1186/s12876-022-02540-2 (PMC9639314; doi:10.1186/s12876-022-02540-2)
Supplement: Supplementary file 4 — Additional file 4: Supplementary Table 3. All the primer sequences. [file 12876_2022_2540_MOESM4_ESM.docx]

**Supplementary table 3 |** All the primer sequences

| **Name** | **Primer sequences (5'to3')** |
| --- | --- |
| NRAV : forward  NRAV : reverse  AC015908.3 : forward | GTTCTTGGCCATCGTGATCT  GGATGAGGTGAGGAGAGCTG  ACAAAGTAGGCAAAGCGGGA |
| AC015908.3 : reverse | ATTCCACCACAGCGTACTCA |
| AC099850.3 : forward | TCGCTATGTTTCCCAGGCTGTATT |
| AC099850.3 : reverse | TGCCAAGGAATCTCTGAAGTCCAT |
| ZFPM2-AS1 : forward | CAATGGGACTAAGCCAGGCA |
| ZFPM2-AS1 : reverse | GGGCTCCACCAACAACCATA |
| GAPDH : forward | ATAGCACAGCCTGGATAGCAACGTAC |
| GAPDH : reverse | CACCTTCTACAATGAGCTGCGTGTG |
|  |  |
